# Humoral responses against SARS-CoV-2 Omicron BA.2.11, BA.2.12.1 and BA.2.13 from vaccine and BA.1 serum

**DOI:** 10.1038/s41421-022-00482-3

**Published:** 2022-11-01

**Authors:** Jiandong Huo, Aiste Dijokaite-Guraliuc, Rungtiwa Nutalai, Raksha Das, Daming Zhou, Alexander J. Mentzer, Elizabeth E. Fry, Juthathip Mongkolsapaya, Jingshan Ren, David I. Stuart, Gavin R. Screaton

**Affiliations:** 1 Division of Structural Biology, Nuffield Department of Medicine, University of Oxford, The Wellcome Centre for Human Genetics, Oxford, UK; 2 State Key Laboratory of Respiratory Disease, National Clinical Research Center for Respiratory Disease, Guangzhou Institute of Respiratory Health, the First Affiliated Hospital of Guangzhou Medical University, Guangzhou, Guangdong, China; 3 Guangzhou Laboratory, Bio-island, Guangzhou, China; 4 Wellcome Centre for Human Genetics, Nuffield Department of Medicine, University of Oxford, Oxford, UK; 5 Chinese Academy of Medical Science (CAMS) Oxford Institute (COI), University of Oxford, Oxford, UK; 6 Oxford University Hospitals NHS Foundation Trust, Oxford, UK; 7 Diamond Light Source Ltd, Harwell Science & Innovation Campus, Didcot, UK

**Keywords:** Immunology, Structural biology

Dear Editor,

Since the outbreak of coronavirus disease 2019 (COVID-19) caused by SARS-CoV-2 in late December 2019 in Wuhan, a pandemic has raged across the world with a series of viral variants emerging in different regions. In November 2021, a variant designated Omicron (BA.1), with over 30 mutations in the Spike protein on the viral surface, was identified in South Africa and quickly became dominant worldwide, due to its increased transmissibility and capacity for immune escape^1^. Thereafter, several sublineages of Omicron have emerged, including BA.2 which has outcompeted BA.1, as well as BA.1.1, BA.3, and BA.4/5^2–4^. Recently, three new variants related to BA.2 have been detected in multiple countries, BA.2.11, BA.2.12.1, and BA.2.13 (named as BA.2 subvariants in the rest of text), which contain a single mutation of L452R, L452Q, or L452M compared to the BA.2 Spike receptor-binding domain (RBD) (https://www.who.int/activities/tracking-SARS-CoV-2-variants) respectively (Supplementary Fig. S1). BA.2.12.1, first identified in New York, became dominant in the US, accounting for about 58% of SARS-CoV-2 isolates as of May 25, 2022^5^. While L452R is found in Delta and Kappa variants, and L452Q in Lambda, L452M is novel. Here we evaluate the receptor binding capacity of the BA.2 subvariants, report a crystal structure of BA.2.12.1 RBD, and show differential sensitivity of these new variants to serum samples and monoclonal antibodies (mAbs) compared to BA.2, providing insights for future vaccine designs.

Considering the physicochemical properties of the side chain of residue 452, BA.2.13 would be expected to be a relatively modest change; L to M will increase the size of the side chain, but it remains hydrophobic. L to Q in BA.2.12.1 introduces some polar character, whilst BA.2.11 is the most radical with L to R introducing a large basic amino acid.

To evaluate the susceptibility of the subvariants to neutralization by immune sera, we performed neutralization assays on pseudotyped lentiviruses expressing the Spike gene of BA.2.11, BA.2.12 and BA.2.13, using a series of serum samples (neutralization curves shown in Supplementary Fig. S1; characteristics of sample donors in Supplementary Table S1). First, we looked at the neutralization profile with sera collected 4 weeks following a third dose of the Oxford-AstraZeneca vaccine AZD1222 (*n* = 41) or Pfizer–BioNtech vaccine BNT162b2 (*n* = 18). No significant loss in neutralization titer was seen compared to BA.2; in fact, despite the L452M mutation being predicted to have a modest effect, BA.2.13 showed a significant increase (1.6-fold, *P* < 0.0001) for AZD1222 vaccinees (Fig. 1a, b). This contrasts with a recent report^4^, where sera collected from triple-dose vaccinees (4 weeks following a third dose of the inactivated vaccine CoronaVac or a ZF2001 booster after two doses of CoronaVac) showed significant reductions in neutralization titer for both BA.2.12.1 and BA.2.13 (BA.2.11 was not tested). Like BA.2, the neutralization profiles of BA.3 are not significantly different from those of the BA.2 subvariants, whilst the titers for BA.4 were substantially lower, due to its additional F486V mutation as previously described^3^.

**Fig. 1:**
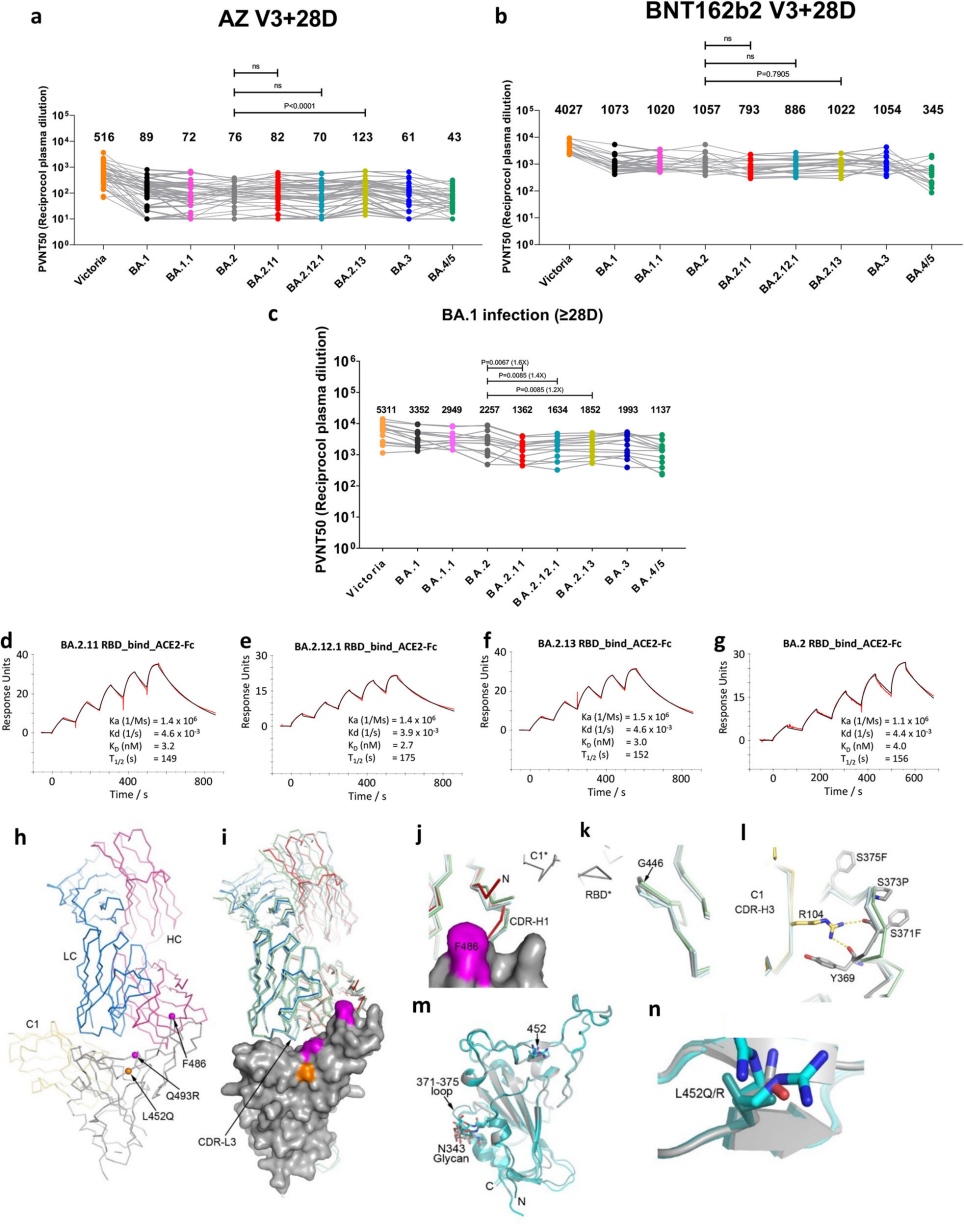
Characterization of BA.2.11, BA.2.12.1 and BA.2.13 by pseudoviral neutralization assays, SPR, and structural analysis. **a**, **b** IC_50_ values for the indicated viruses using serum obtained 4 weeks after a third dose of vaccine (**a**) AstraZeneca AZD1222 (*n* = 41), **b** Pfizer BNT162b2 (*n* = 18). **c** Neutralization titers of serum from vaccinated volunteers suffering breakthrough BA.1 infection. Comparison is made with neutralization titers to Victoria (SARS-CoV-2/human/AUS/VIC01/2020), BA.1, BA.1.1, BA.2, BA.3, and BA.4/5 as previously reported^3^. Geometric mean titers are shown above each column. The Wilcoxon matched-pairs signed-rank test was used, and two-tailed *P* values calculated. **d**–**g** SPR sensorgrams (red: experimental binding curve; black: fitted curve) showing ACE2 binding of RBD of BA.2.11 (**d**), BA.2.12.1 (**e**), BA.2.13 (**f**) in comparison with binding to BA.2 RBD (**g**) (kinetics data shown). Data for BA.2 RBD were reported previously^2^. **h**–**n** Crystal structure of BA.2.12.1 RBD/Beta-27/NbC1 complex. Cα traces with RBD (gray), Beta-27 HC (red) and LC (blue), and NbC1 (yellow). Cαs of L452Q, F486 and Q493R (L, F, and R in BA.2, R, V, and Q in BA.4/5, respectively) shown as spheres (**h**). Comparison of Beta-27 binding modes between BA.2.12.1 RBD/Beta-27/NbC1 (RBD as surface representation, HC red and LC blue), BA.4/5 RBD/Beta-27/NbC1 (cyan, PDB 7ZXU) and Beta RBD/Beta-27 (green, PDB 7PS1) complexes (RBDs are superposed). Apart from the flexible N- and C-terminal regions of RBD, significant differences occur at the N-terminus and CDR-H1 of the Fab HC, α2 helix, 371–375 loop, and G446 loop of the RBD. CDR-L3 has two conformations in the BA.4/5 RBD complex, and one in other complexes (**i**). The HC N-terminus and CDR-H1 which contacts RBD residue 486 differs in both Beta and BA.4/5 RBD complexes, the later contains the F486V mutation. Differences are likely caused by contacts from a symmetry-related C1 nanobody shown as gray bonds (**j**). A structural difference in the G446 loop in the BA.4/5 RBD is induced by crystal contacts (**k**). The 371–375 loop carrying S371F, S373P, and S375F mutations in BA.2.12.1 and BA.4/5 RBDs is stabilized by interactions with NbC1 CDR-H3 (**l**). Superimposition of BA.2.12.1 (gray), BA.2 (green, PDB 7ZF9), and BA.4/5 (cyan) RBDs (**m**). 452 mutations do not introduce significant local structural changes. R452 in BA.4/5 has two conformations (**n**).

Next, we examined the neutralization profile for serum samples collected from vaccinees infected with BA.1. Samples (*n* = 14) were taken ≥28 days following symptom onset (median 38 days); all convalescent individuals had two doses of vaccine before BA.1 infection, and four of them had a third dose following BA.1 infection and prior to sample collection (Supplementary Table S1). There were significant reductions in neutralization titer for all three subvariants compared to BA.2, with the greatest decrease for BA.2.11 (1.6-fold, *P* = 0.0067), followed by BA.2.12.1 (1.4-fold, *P* = 0.0085) and BA.2.13 (1.2-fold, *P* = 0.0085) (Fig. 1c). Together, these observations suggest that, compared with BA.2, its subvariants are not showing stronger humoral immune escape in individuals recently vaccinated with three doses of AZD1222 or BNT162b2. However, for vaccinees who had a BA.1 breakthrough infection, regardless of the type of vaccine they received, the BA.2 variants are more capable of evading the humoral response, although a broad neutralizing antibody response, with high titers to all variants of concern, is induced^2^. This may indicate the different selective pressure on BA.2 and its subvariants on a high background of breakthrough infection. As antibody titers naturally wane at longer time points, people with BA.1 breakthrough infection are expected to be more susceptible to reinfection with the BA.2 subvariants.

To further elucidate the differential responses between BA.2 and its subvariants, we performed pseudoviral assays on a panel of potent human monoclonal antibodies (mAbs) generated from cases of BA.1 breakthrough infection^2^ (Supplementary Fig. S2 and Table S2). In line with the structural inference and serum neutralization results the greatest reduction of mAb neutralization titer was for BA.2.11, followed by BA.2.12.1 and BA.2.13. BA.2.11 neutralization of BA.2.11 completely knocked out for 5/27 mAbs (Omi-06, Omi-24, Omi-30, Omi-31, and Omi-34). In a previous study^2^, we reported that Omi-06 (an IGHV4-4 mAb) has a similar binding footprint to Omi-24/30/31/34 (IGHV1-69 mAbs) which are positioned close to residue 452. This explains why these five mAbs have similar sensitivity profiles to both Delta and BA.2.11 which bear the same L452R mutation. The neutralizing activity against BA.2.12.1 was also reduced to varying degrees for the same set of mAbs, whilst the profiles were largely unchanged against BA.2.13. Among them, Omi-06 belongs to the IGVH4-4 family, and the remainder to the IGVH1-69 family. Indeed, previous structural studies predicted Omi-06 and Omi-31 to be sensitive to the L452R mutation in Delta^2^. To confirm that the differential neutralization effects observed are directly attributable to the changes in RBD binding, we used surface plasmon resonance (SPR) to compare the binding behavior of BA.2 and BA.2.12.1 RBD, using Omi-06 and Omi-31 as examples. As expected, the affinities were reduced, for Omi-06, BA.2.12.1 RBD was 15-fold weaker binding than BA.2 and, strikingly, the binding of BA.2.12.1 RBD to Omi-31 was ~1300-fold weaker (Supplementary Fig. S3).

To evaluate the possible change in transmissibility of the BA.2 subvariants we performed SPR experiments to analyze their RBD binding to ACE2 (Fig. 1d–g). The three RBD variants have an affinity of ~3 nM for ACE2, a little higher than that of BA.2 RBD (*K*_D_ = 4 nM) as previously reported^2^. Modeling of the ACE2/RBD complex suggests that this may result from slightly improved complementarity between ACE2 and the RBD due to the leucine 452 mutation. It is unclear whether these small changes in affinity would influence transmissibility.

To verify structural inferences, we determined the structure of BA.2.12.1 RBD at 2.38 Å (Supplementary Table S3) as a ternary complex with nanobody NbC1^6^, which interacts away from the ACE binding site in a conserved region and the Fab of neutralizing mAb Beta-27^7^ belonging to the public antibody IGHV3-53 family which binds, as expected, at the back of the neck of the RBD, overlapping the ACE2 binding site (Fig. 1h–n). Structural differences are essentially restricted to the side-chain of residue 452 (RMSD in RBD Cα positions vs BA.2 [PDB:7ZF7]: 0.8 Å).

In summary, we show that Spike mutations in the newly emerged BA.2 variants could render it slightly more transmissible than BA.2. However, compared to BA.2, they do not appear to have acquired greater humoral immune escape in healthy vaccinees who have received three doses of the Oxford-AstraZeneca or Pfizer–BioNtech BNT162b2 vaccine. This result differs from that of vaccinees who have received the triple-dose CoronaVac vaccine, for whom significantly reduced neutralization titers were observed. A surprising and unexplained increase in BA.2.13 neutralizing titers compared to BA.2 was observed for AZD1222^4^. Nevertheless, neutralization titers were significantly reduced in vaccinees who had experienced BA.1 breakthrough infections, no matter which type of vaccine was received, perhaps partly due to partial or complete knockout of neutralizing activity of antibodies belonging to the IGVH1-69 family, many of which are sensitive to the mutation at leucine 452 of the RBD. This suggests that the continuously evolving Omicron sublineages are able to gain evasion from the humoral immune responses mounted by BA.1, implying that BA.1 Spike or RBD might not be a substantially better immunogen than that of the ancestral Wuhan strain for the development of the next-generation SARS-CoV-2 vaccine.

## Supplementary information

Supplementary Information
