# Supplementary material for: Humoral responses against SARS-CoV-2 Omicron BA.2.11, BA.2.12.1 and BA.2.13 from vaccine and BA.1 serum
Source: Cell Discov. 2022 Nov 1;8:119. doi: 10.1038/s41421-022-00482-3 (PMC9626457; doi:10.1038/s41421-022-00482-3)
Supplement: Supplementary file 1 — Supplementary Information [file 41421_2022_482_MOESM1_ESM.pdf]

Supplementary Fig. S1: Neutralization curves for serum samples.

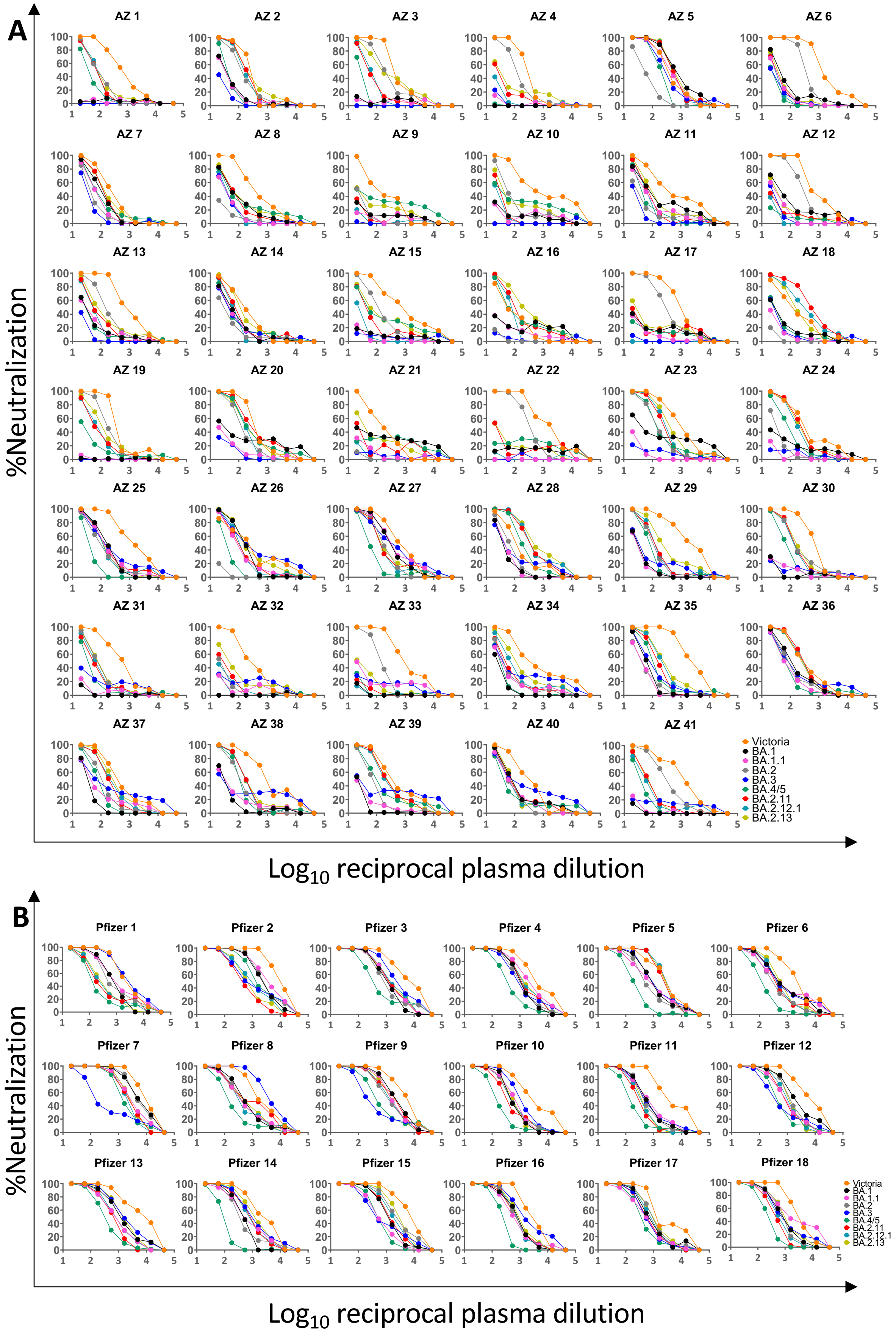

Supplementary Fig. S1: Neutralization curves for serum samples.

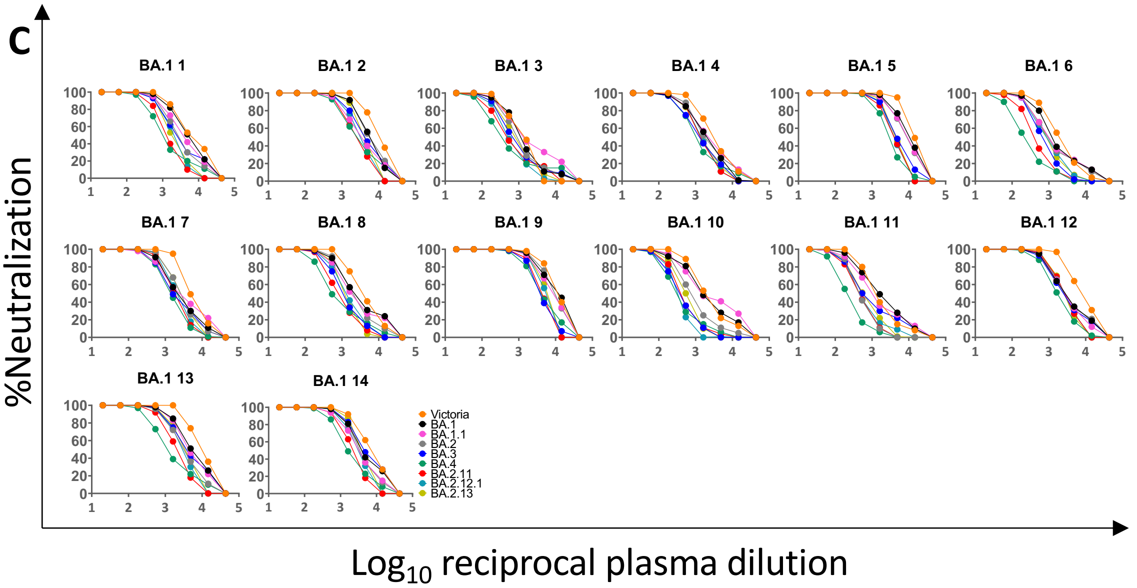

Supplementary Fig. S1 Pseudoviral neutralization assays against different serum samples, related to Figure 1. a-c where IC50 titres are shown. Neutralization curves for a panel of serum samples against different SARS-CoV-2 variants.

Supplementary Fig. S2: Pseudoviral neutralization assays against Omicron monoclonal antibodies, related to Supplementary Table S1 where IC50 titres are shown.

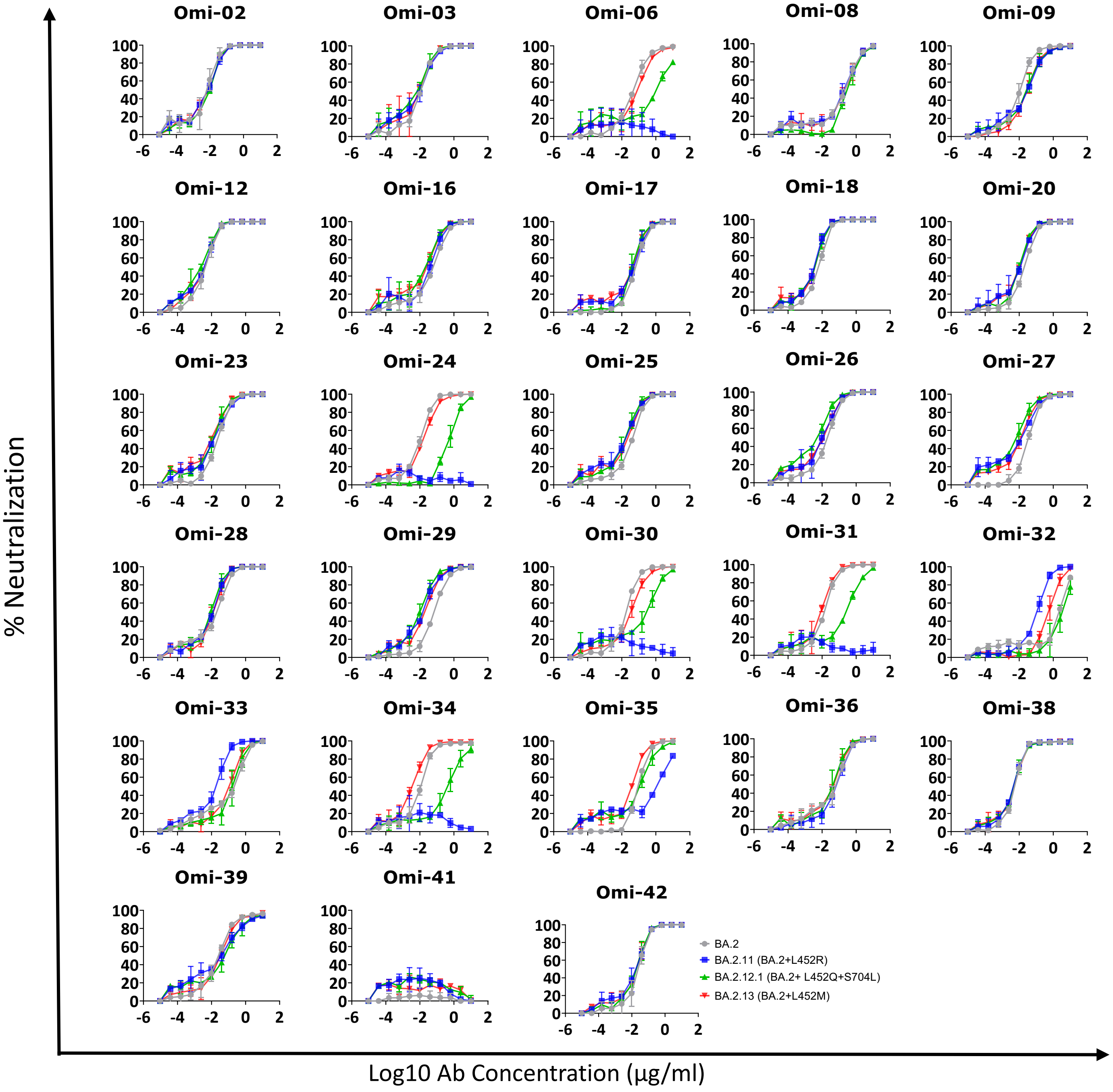

Supplementary Fig. S2 Pseudoviral neutralization assays against Omicron monoclonal antibodies, related to Supplementary Table S1 where IC50 titres are shown. Neutralization curves for a panel of 27 monoclonal antibodies made from samples taken from vaccinees infected with BA.1. Titration curves for BA.2.11, BA.2.12.1 and BA.2.13 are compared with BA.2.

Supplementary Fig. S3: Surface plasmon resonance (SPR) analysis of the interaction between BA.2.12.1 or BA.2 RBD and selected mAbs (Omi-6 and Omi-31).

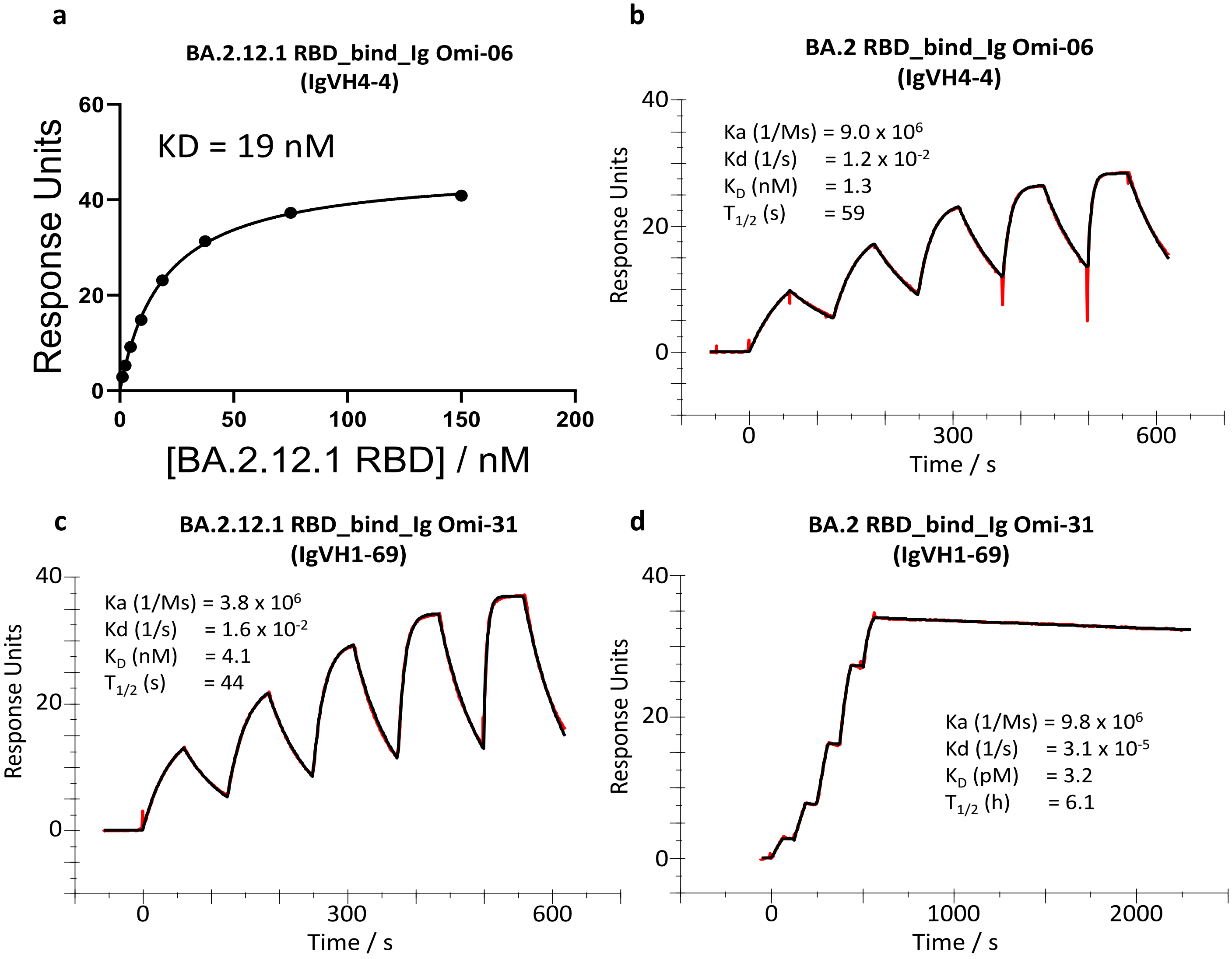

Supplementary Fig. S3: Surface plasmon resonance (SPR) analysis of the interaction between BA.2.12.1 or BA.2 RBD and selected mAbs (Omi-6 and Omi-31). **a** Determination of the affinity of BA.2.12.1 RBD to Omi-6 using a 1:1 binding equilibrium analysis. **b, c, d** Sensorgrams (red: original binding curve; black: fitted curve) showing the interactions between BA.2.12.1 or BA.2 RBD and selected mAbs, with kinetics data shown.

Supplementary Table S1. Characteristics of participants

**AZ V3+28**	
**Participants**	
Female	20
Male	21
**Average Age (Y)**	37 (Range 25-53)
**Vaccine History**	
First vaccine	ChAdOx1 (2020 Apr-2020 May)
Second vaccine	ChAdOx1 (2020 Jul-2020 Aug)
Third vaccine	ChAdOx1 (2021 March)
**Infection History**	None
**Days post last vaccination (D)**	28

**BNT162b2 V3+28**	
**Participants**	
Female	8
Male	10
**Average Age (Y)**	45 (Range 30-59)
**Vaccine History**	
First vaccine	BNT162b2 (2020 Dec-2021 Jan)
Second vaccine	BNT162b2 (2021 Jan)
Third vaccine	BNT162b2 (2021 Sep-2021 Nov)
**Infection History**	None
**Days post last vaccination (D)**	28

**BA.1 infection**	
**Participants**	
Female	7
Male	7
**Average Age (Y)**	29 (Range 21-56)
**Vaccine History**	
First dose	2020 Dec-2021 Jul
ChAdOx1	4
BNT162b2	9
J&J	1
Second dose	2021 Jan-2021 Sep
ChAdOx1	4
BNT162b2	9
Third dose	2021 Sep-2022 Jan
BNT162b2	11
**Infection History**	BA.1 (2021 Aug-2021 Dec)
Pre-3rd dose	4
Post-vaccine (1 dose)	1
Post-vaccine (2 doses)	2
Post-vaccine (3 doses)	7
**Average days after infection (D)**	42 (IQR 27-55)

Supplementary Table S2. IC50 values for Omicron mAbs

mAb	IC50 (μg/ml)			
	BA.2	BA.2.11	BA.2.12.1	BA.2.13
Omi02	0.003 ± 0.000	0.004 ± 0.001	0.005 ± 0.001	0.004 ± 0.000
Omi03	0.008 ± 0.001	0.005 ± 0.002	0.003 ± 0.001	0.007 ± 0.005
Omi06	0.039 ± 0.008	>10	0.616 ± 0.123	0.046 ± 0.024
Omi08	0.114 ± 0.045	0.099 ± 0.020	0.358 ± 0.076	0.117 ± 0.009
Omi09	0.008 ± 0.002	0.016 ± 0.005	0.015 ± 0.003	0.022 ± 0.002
Omi12	0.003 ± 0.001	0.002 ± 0.000	0.001 ± 0.000	0.003 ± 0.000
Omi16	0.034 ± 0.012	0.017 ± 0.004	0.011 ± 0.005	0.008 ± 0.000
Omi17	0.060 ± 0.004	0.022 ± 0.008	0.034 ± 0.001	0.016 ± 0.001
Omi18	0.005 ± 0.000	0.002 ± 0.000	0.002 ± 0.000	0.002 ± 0.001
Omi20	0.015 ± 0.003	0.007 ± 0.004	0.007 ± 0.000	0.006 ± 0.000
Omi23	0.019 ± 0.005	0.009 ± 0.003	0.006 ± 0.002	0.005 ± 0.001
Omi24	0.007 ± 0.001	>10	0.450 ± 0.140	0.008 ± 0.000
Omi25	0.024 ± 0.004	0.007 ± 0.001	0.009 ± 0.002	0.010 ± 0.000
Omi26	0.013 ± 0.001	0.007 ± 0.003	0.002 ± 0.000	0.006 ± 0.000
Omi27	0.034 ± 0.006	0.005 ± 0.001	0.003 ± 0.001	0.006 ± 0.000
Omi28	0.008 ± 0.000	0.007 ± 0.000	0.005 ± 0.000	0.009 ± 0.001
Omi29	0.056 ± 0.014	0.011 ± 0.001	0.007 ± 0.001	0.012 ± 0.001
Omi30	0.013 ± 0.002	>10	0.086 ± 0.026	0.020 ± 0.002
Omi31	0.011 ± 0.002	>10	0.089 ± 0.035	0.008 ± 0.004
Omi32	2.614 ± 0.533	0.070 ± 0.008	4.988 ± 0.080	0.503 ± 0.080
Omi33	0.070 ± 0.024	0.008 ± 0.002	0.086 ± 0.045	0.055 ± 0.007
Omi34	0.009 ± 0.003	>10	0.408 ± 0.140	0.003 ± 0.001
Omi35	0.092 ± 0.004	0.667 ± 0.104	0.188 ± 0.074	0.016 ± 0.004
Omi36	0.030 ± 0.014	0.051 ± 0.027	0.026 ± 0.011	0.020 ± 0.004
Omi38	0.005 ± 0.000	0.004 ± 0.001	0.003 ± 0.000	0.003 ± 0.001
Omi39	0.026 ± 0.011	0.018 ± 0.003	0.068 ± 0.008	0.025 ± 0.007
Omi42	0.021 ± 0.011	0.009 ± 0.003	0.012 ± 0.001	0.009 ± 0.001

Supplementary Table S3. X-ray data collection and structure refinement statistics

[a] Values in parentheses are for highest-resolution shell.

**Structure**	BA.2.12.1 RBD/Beta-27/NbC1
**Data collection**	
Space group	C2
Cell dimensions	
a, b, c (Å)	186.8, 100.0, 56.5
a, b, g (°)	90, 104.1, 90
Resolution (Å)	55–2.38 (2.42–2.38)[a]
R_{merge}	0.240 (---)
R_{pim}	0.071 (1.366)
I/s(I)	6.3 (0.3)
$CC_{1/2}$	0.988 (0.13)
Completeness (%)	94.8 (67.7)
Redundancy	11.2 (4.4)
**Refinement**	
Resolution (Å)	55–2.38
No. reflections	35221/1842
R_{work} / R_{free}	0.186/0.233
No. atoms	
Protein	5723
Ligand/ion/water	259
B factors ($Å^2$)	
Protein	58
Ligand/ion/water	60
r.m.s. deviations	
Bond lengths (Å)	0.002
Bond angles (°)	0.5

Supplementary Table S4. Primer sequences used to generate pseudoviruses. Related to Plasmid construction and pseudotyped lentiviral particle production.

Primer	Sequence (5’to 3’)
**BA.2.11**	
L452R_F	GGAGGCAATTACAATTACCGGTACAGACTGTTCAGAAAG
L452R_R	CTTTCTGAACAGTCTGTACCGGTAATTGTAATTGCCTCC
**BA.2.12.1**	
L452Q_R	CTTTCTGAACAGTCTGTACTGGTAATTGTAATTGCCTCC
L452Q_F	GGAGGCAATTACAATTACCAGTACAGACTGTTCAGAAAG
S704L_F	GAGCCTGGGCGCCGAGAATCTAGTGGCCTACAGCAATAATAG
S704L_R	CTATTATTGCTGTAGGCCACTAGATTCTCGGCGCCCAGGCTC
**BA.2.13**	
L452M_F	GTTGGAGGCAATTACAATTACATGTACAGACTGTTCAGAAAGA
L452M_R	TCTTTCTGAACAGTCTGTACATGTAATTGTAATTGCCTCCAAC
